# Recent Advances in the Synthetic Biology of Natural Drugs

**DOI:** 10.3389/fbioe.2021.691152

**Published:** 2021-07-29

**Authors:** Chun-Qiang Li, Hong-Mei Lei, Qian-Yi Hu, Guo-Hong Li, Pei-Ji Zhao

**Affiliations:** State Key Laboratory for Conservation and Utilization of Bio-Resources in Yunnan, and Key Laboratory for Microbial Resources of the Ministry of Education, Yunnan University, Kunming, China

**Keywords:** natural drugs, synthetic biology, biosynthesis, expression system, optimization

## Abstract

Natural drugs have been transformed and optimized during the long process of evolution. These compounds play a very important role in the protection of human health and treatment of human diseases. Sustainable approaches to the generation of raw materials for pharmaceutical products have been extensively investigated in drug research and development because chemical synthesis is costly and generates pollution. The present review provides an overview of the recent advances in the synthetic biology of natural drugs. Particular attention is paid to the investigations of drugs that may be mass-produced by the pharmaceutical industry after optimization of the corresponding synthetic systems. The present review describes the reconstruction and optimization of biosynthetic pathways for nine drugs, including seven drugs from plant sources and two drugs from microbial sources, suggesting a new strategy for the large-scale preparation of some rare natural plant metabolites and highly bioactive microbial compounds. Some of the suggested synthetic methods remain in a preliminary exploration stage; however, a number of these methods demonstrated considerable application potential. The authors also discuss the advantages and disadvantages of the application of synthetic biology and various expression systems for heterologous expression of natural drugs. Thus, the present review provides a useful perspective for researchers attempting to use synthetic biology to produce natural drugs.

## Introduction

Natural medicines are natural products with pharmacological activity. Most of these products are compounds produced by secondary metabolism in animals, plants, and microorganisms (Bernardini et al., 2018; Newman and Cragg, 2020). These natural products have been transformed and optimized during the long process of evolution and have good medicinal value (Chevrette et al., 2019; Karunanithi and Zerbe, 2019). These products play very important roles in the protection of human health and in the treatment of human diseases. From 1981 to 2019, 59% of newly approved small molecule drugs (a total of 1,123) were derived from natural compounds and their derivatives; thus, natural compounds are important resources for modern drug development (Morrison and Hergenrother, 2014; Newman and Cragg, 2020). However, recent demands for drug production and quantity have increased due to increased resistance to pathogenic microorganisms, and traditional methods of the production of natural medicines are unable to meet these demands. The sustainability and green development of production are becoming increasingly prominent needs. Synthetic biology is a new type of modern interdisciplinary life science and systems science that has emerged in the twenty-first century that combines traditional metabolic engineering and concepts of systems biology. Recent applications of synthetic biology have provided a strong stimulus to study about natural medicines. Optimized and transformed organisms can continuously and efficiently synthesize specific target compounds with high yield and are characterized by low carbon emissions, environmental friendliness, and cost effectiveness. There is no need to rely solely on traditional discovery and separation or complex synthetic chemistry to obtain a limited number of natural drugs and analogs because new biosynthetic pathways can be designed to produce numerous natural drugs and analogs (Nielsen and Keasling, 2011; Frasch et al., 2013). Development of synthetic biology may bring natural drug research into a new era.

The technology and methods of the synthetic biology of natural products have been extensively reviewed, and basic design principles, routes, and optimization methods have been proposed (Moses et al., 2017; Cravens et al., 2019). The present review describes some studies on natural plant drugs and microbial drugs reported in the past decade and discusses the results of these studies, focusing on the advantages and disadvantages of various heterologous expression systems (*Escherichia coli, Bacillus subtilis*, yeast, and filamentous fungi). Thus, the present review provides a useful perspective for researchers who plan to use synthetic biology to produce natural drugs.

## Application of Synthetic Biology to Produce Natural Drugs From Plants

**Figure 1:**
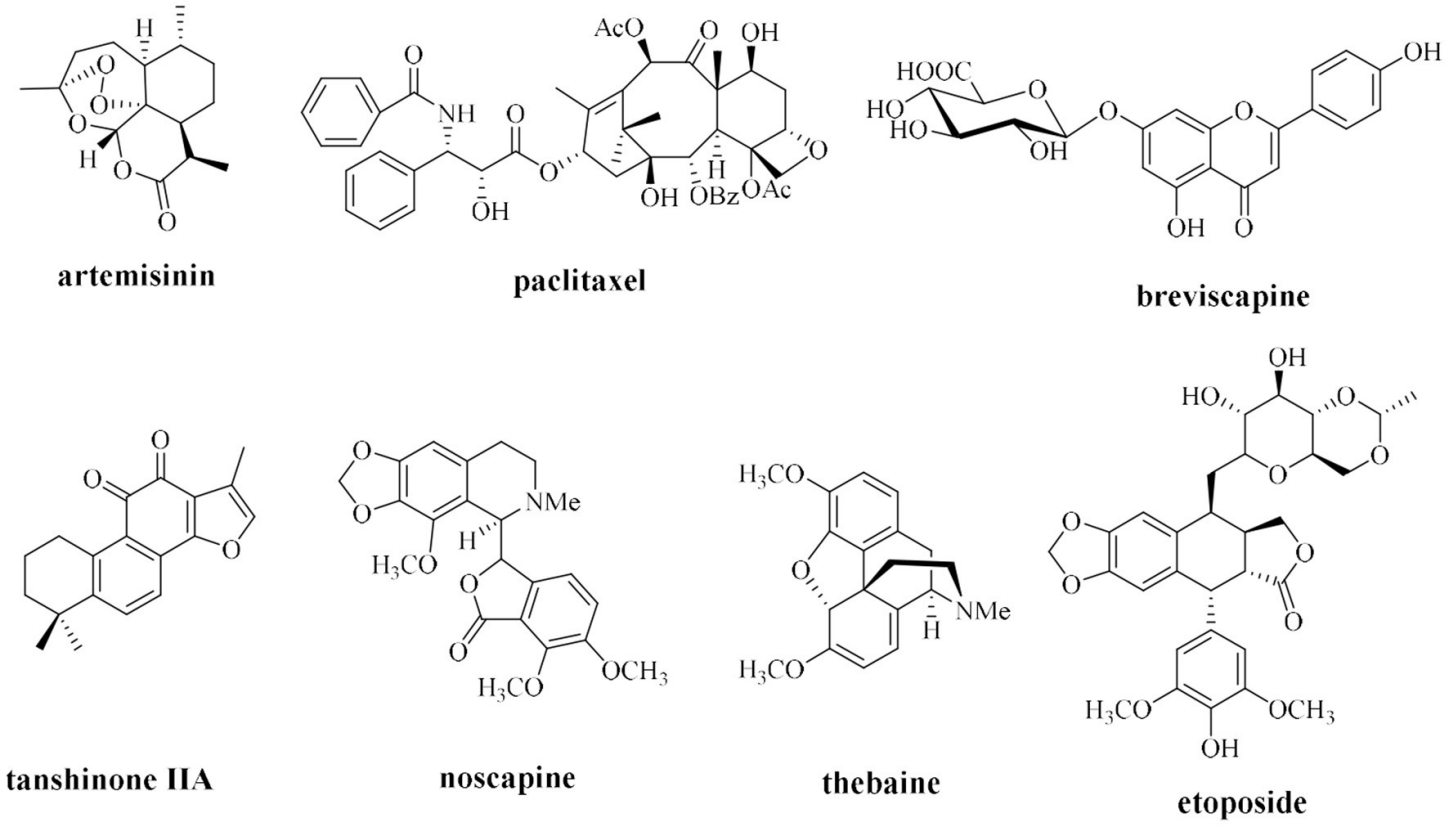
Some natural drugs studied by synthetic biology.

Currently, plant extraction is a primary method of the production of natural drugs from plants; however, traditional methods of preparation of natural compounds that rely on plant extraction have many limitations. Most natural plant medicines are commonly characterized by extremely low content in the host; for example, ~3 kg of bark of a 100-year-old Pacific yew may contain only 300 mg of paclitaxel, accounting for ~0.01% of the dry weight of the bark (Horwitz, 1994). The content of the active ingredient ginsenoside in ginseng aged from 3 to 5 years accounts for only ~2% of the dry weight of the root, and the content of some rare saponins with important medicinal activities is ~0.02–0.0009 wt% in dried roots (Yoshikawa et al., 1997; Liu, 2012). Traditional plant extraction methods require isolation of trace active ingredients from large quantities of plant resources, which causes considerable waste and serious damage to wild plant resources and may threaten some endangered plant species. Thus, the biosynthesis of natural medicines, such as artemisinin, paclitaxel, tanshinone, breviscapine, noscapine, and thebaine, is used as an example (Figure 1) to apply research advances in synthetic biology to the synthesis of natural plant compounds.

### Production of Artemisinin by Combining Synthetic Biology and Synthetic Chemistry

Malaria is one of the most severe parasitic diseases worldwide. More than 214 million people were infected with malaria in 2015, leading to 438,000 deaths, primarily among children under 5 years of age. Artemisinin-based combination therapies are currently considered by WHO to be the most effective method for treating malaria (Banek et al., 2014). In 1972, the Chinese scientist Tu and her team obtained a colorless crystal compound named artemisinin from the plant *Artemisia annua* (Tu, 2011). However, the content of artemisinin in *A. annua* accounts for only 0.1–1.0% of the dry weight of the plant. The production cost of artemisinin is very high due to the unstable structure of artemisinin and complexity of the purification process and hampers the efforts to meet the market demands. Therefore, identification of a more efficient route for the production of artemisinin is urgently needed.

**Figure 2:**
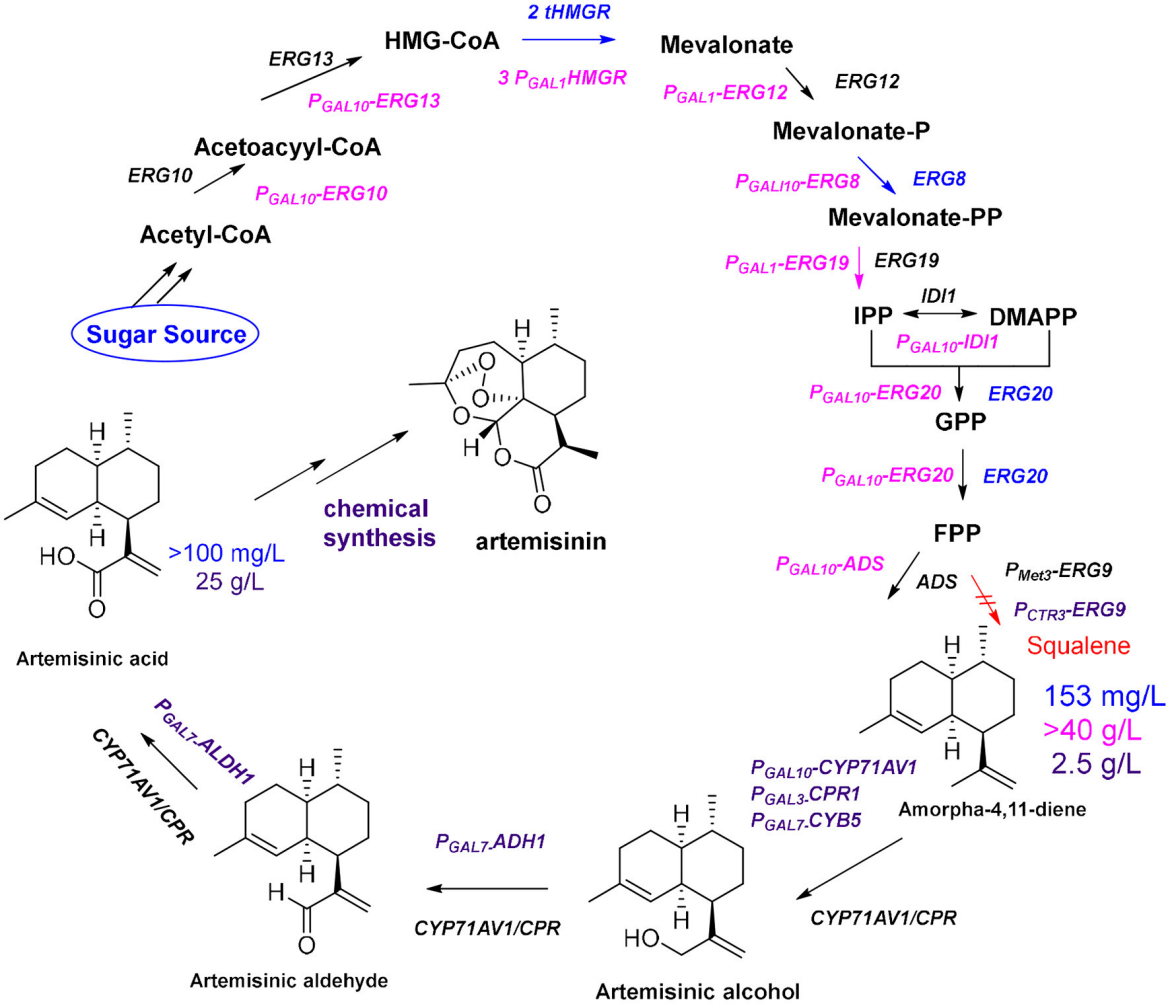
Optimization of the production of artemisinin**. Blue route:** By enhancing the expression of *tHMGR* and *ERG20*, together with *ADS* and *CYP71AV1/CPR*, the yields of amorpha-4,11-diene and artemisinic acid are 153 mg/L and more than100 mg/L, respectively**; Pink route:** After using *S. cerevisiae* CEN.PK2 as chassis and introducing a strong promoter (*GAL*_1_ or *GAL*_10_) before each gene (*ERG10, ERG13, tHMG1, ERG12, ERG8, ERG19, IDI1*, and *ERG20*), the yield of amorpha-4,11-diene reaches 40 g/L; **Purple route:** By enhancing expression two genes (ADH1 and ALDH1) and using the *CTR3* promoter suppress expression of *ERG9*, the yield of artemisinic acid reached 25 g/L after optimizing fermentation conditions.

The development of high-throughput sequencing and molecular technology has led to the elucidation of the biosynthetic pathway for artemisinin, and the genes of some key enzymes of the biosynthetic pathway of artemisinin have been cloned and characterized (Lu et al., 2013). The universal 5-carbon precursors isopentenyl pyrophosphate (IPP) and its double-bond isomer dimethylallyl pyrophosphate (DMAPP) are derived from the mevalonate pathway (MVA) in the cytoplasm of *A. annua* and from the methylerythritol-4-phosphate pathway (MEP) in plastids (Ma et al., 2015). The artemisinin precursor farnesyl diphosphate (FPP) combines two IPP molecules and a single DMAPP molecule and was shown to be involved in the formation of various isoprenoids (artemisinin, aristolochene, caryophyllene, farnesene, and sterols). The formation of FPP is catalyzed by farnesyl diphosphate synthase (FPS) in artemisinin-producing organisms (Abdin and Alam, 2015). Amorpha-4,11-diene (AD) is the first specific sesquiterpenoid precursor of the artemisinin biosynthesis pathway, formed by cyclization of FPP catalyzed by amorpha-4,11-diene synthase (ADS) (Nguyen et al., 2011). In 2006, the Keasling group (Ro et al., 2006) cloned the amorpha-4,11-diene oxidase gene CYP71AV1 from the glandular hairs of *A. annua* and simultaneously introduced ADS, CYP71AV1, and CPR into *Saccharomyces cerevisiae*. The yield of artemisinic acid in this yeast cell factory was more than 100 mg/L (Figure 2, blue route). Artemisinin acid can be efficiently and inexpensively converted to artemisinin by semi-chemical synthesis.

To further improve the yield of artemisinic acid, all genes required for FPP synthesis were overexpressed in *S. cerevisiae* CEN.PK2 (Westfall et al., 2012), including *ERG10, ERG13, tHMG1, ERG12, ERG8, ERG19, IDI1*, and *ERG20*, and three copies of *tHMG1* were integrated to reach the 40 g/L fermentation level of the artemisinin precursor amorpha-4,11-diene (Figure 2, pink route). Moreover, the Keasling group (Paddon et al., 2013) combined the expression of *ADH1* (the gene encoding NAD alcohol dehydrogenase), *ALDH1* (the gene encoding NAD acetaldehyde dehydrogenase), and *CYB5* (the cytochrome b5 gene) to produce the highest concentration of artemisinic acid (25 g/L; Figure 2, purple route). In the final steps of artemisinin biosynthesis, it is still unclear whether the conversion from dihydroartemisinic acid to artemisinin is enzymatic or nonenzymatic in *A. annua*. It is known that dihydroartemisinic acid could be converted to artemisinin in a non-enzymatic manner through spontaneous autoxidation (Sy and Brown, 2002). However, it is not clear whether a series of similar spontaneous oxidations occurs in plants, or whether there are enzymes to catalyze certain steps in this pathway *in vivo* (Brown and Sy, 2004; Bryant et al., 2015). In general, many biosynthetic pathways in *A. annua* competes with the artemisinin biosynthetic pathway for metabolic flux; therefore, RNA interference technology is used to introduce antisense strands of cDNA into *A. annua* to downregulate the expression levels of β-farnesyl pyrophosphate synthase, β-caryophyllene synthase (CPS), and squalene synthase to block the effects of the pathways that compete with artemisinin biosynthesis (Lv et al., 2016). The yield of artemisinin in all transgenic plants of *A. annua* increased by ~70% compared with that obtained from the control plants. In addition, organism is a complex membrane structure system; compartmentalization of secondary metabolite biosynthetic process not only allows enzymes to be aggregated as functional units, but also separates the pathway from the rest of the cell. Co-compartmentalization of biosynthetic enzymes can be expected to allow certain types of selective advantages. The colocalization of continuous enzymes in the secondary metabolite biosynthetic pathway can promote pathway efficiency through proximity. This is especially important if the products and intermediates may be toxic to the production cell (Roze et al., 2011; Kistler and Broz, 2015). In *A. annua*, artemisinin biosynthesis is localized in the two outer apical cells of the glandular trichomes, which are specialized structures, found mainly on the surface of leaves and flowers (Olsson et al., 2009). Furthermore, oxidation reactions of dihydroartemisinic acid are also believed to occur in the glandular trichomes (Brown, 2010). But how this special structure plays a role in oxidation catalysis is still unclear. In 2016, the Fuentes team (Fuentes et al., 2016) developed a method of combinatorial super transformation of chloroplast-transformed recipient lines (COSTREL), which constructed large transformation vectors expressing multiple pathway genes, and inserted them into chloroplast genomes. They screened many transgenic lines by their physiological and phenotypic changes. This strategy transferred the entire biochemical metabolic pathway from a medicinal plant to a high-biomass crop, and 120 mg of artemisinic acid was isolated per kilogram of transgenic tobacco. A complete pathway of artemisinin synthesis was achieved in tobacco by coexpressing five genes [*HMGR, ADS, CYP71AV1, CPR*, and *DBR2* (the gene encoding artemisinic aldehyde double bond reductase)] and the artemisinin content reached 6.8 μg/g dry weight (Farhi et al., 2011a). Through further optimization of metabolic pathways and using cellular compartments, the yield of artemisinin in tobacco was increased to 0.8 mg/g dry weight (Malhotra et al., 2016). This yield is much lower than the content of artemisinin in *A. annua*, but it is an important achievement. In general, metabolic optimization can increase the content of target compounds, but how cellular compartments work is unclear. A specific lipid transfer protein from *A. annua* can boot transient production of artemisinin in tobacco (Wang et al., 2016). This result further suggested that compartment plays an important role in artemisinin biosynthesis (Ikram et al., 2017) because the function of transporters is to transport specific molecules to particular areas *in vivo*. In plants the metabolic networks are compartmentalized, and biochemical steps of a single pathway can occur in multiple subcellular locations. Due to the results of tracking the distribution of proteins and metabolites in the various compartments, we have a certain understanding of the reactions and precursor compounds in various compartments (Heinig et al., 2013). Up to now, the main source of artemisinin on the market is still the extract from *A. annua*. An alternative approach would be to try to reconstruct the microbial fermentation process by mimicking the process that occurs under natural conditions. This requires us to deeply understand the functions and mechanisms of specific compartments in metabolic networks. For example, how dihydroartemisinic acid is oxidized to artemisinin in glandular trichomes, which requires more fundamental research. But the ultimate reward will be the production of artemisinin through a single fermentation process.

### Production of the Paclitaxel Precursors by Pathway Optimization

Paclitaxel is a diterpenoid anticancer drug isolated from Chinese yew (*Taxus chinensis*) and is the first-line drug for the clinical treatment of ovarian cancer and breast cancer (Zhang, 2014). The content of paclitaxel in yew is very low, and the resources of *T. chinensis* are scarce. The route of total chemical synthesis of paclitaxel is complex and costly; therefore, semichemical synthesis is an important method for the industrial production of paclitaxel (Li et al., 2015). The basic biosynthetic route of paclitaxel has been determined, and most of the enzymes have been obtained and functionally identified. Currently, 14 of the 19 enzymes required for the biosynthetic pathway of paclitaxel have been identified; however, the order in which most of these enzymes, especially P450 enzymes, participate in the pathway has not yet been determined (Jennewein and Croteau, 2001; Schoendorf et al., 2001; Jennewein et al., 2005; Koeksal et al., 2011; Wang et al., 2018).

**Figure 3:**
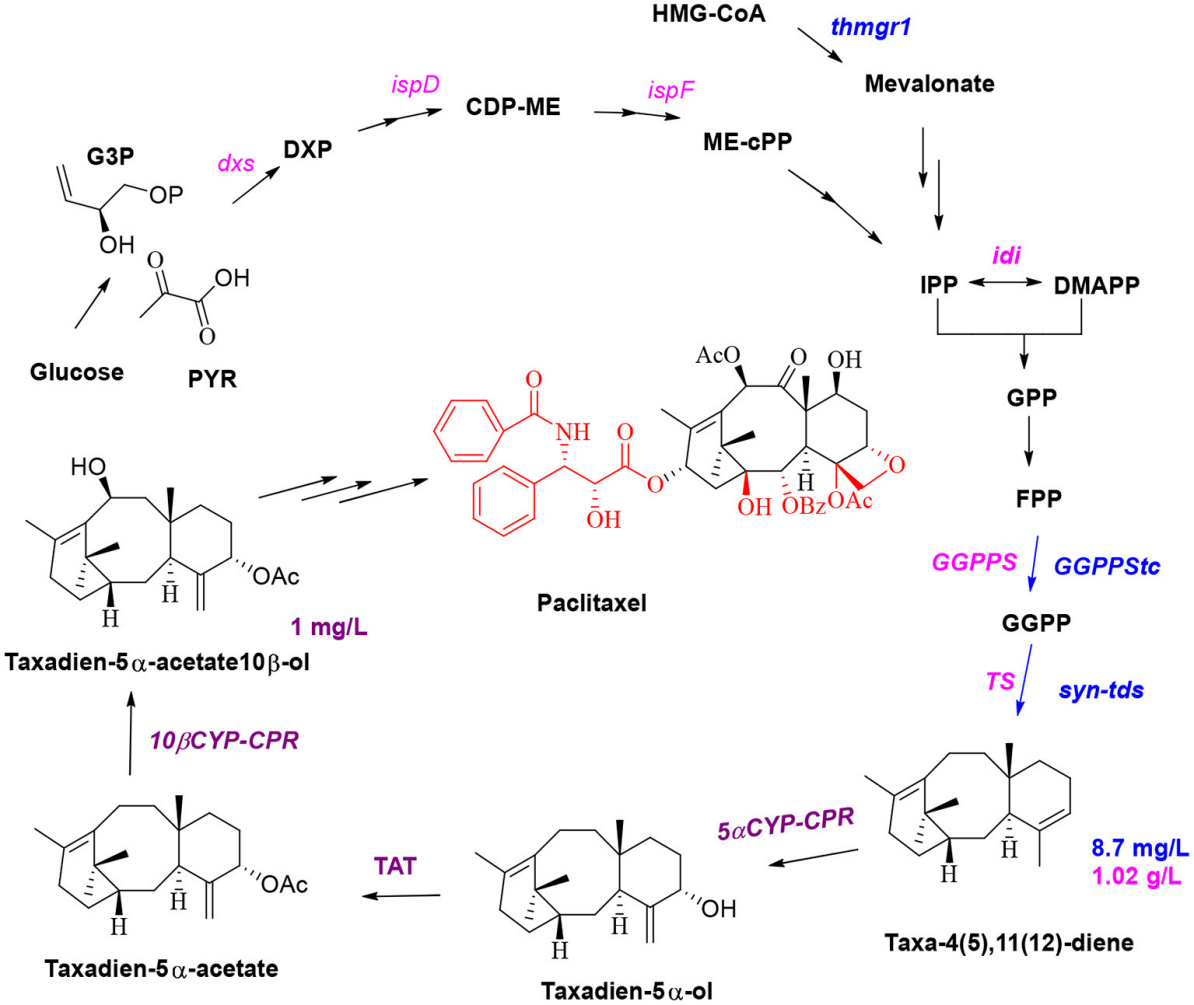
Optimization of the production of the paclitaxel precursors.

Microbial synthesis of paclitaxel precursors and semisynthesis of paclitaxel are currently the most promising methods for the production of this drug, and these methods are expected to solve the problems of high price and short supply of paclitaxel in the market. It is also very important to protect endangered *T. chinensis* (Liu et al., 2016). A brief biosynthetic pathway involves the cyclization of GGPP to taxa-4(5),11(12) diene by taxadiene synthase (Koeksal et al., 2011); hydroxylation of taxane skeleton at C5, C10, C13, C2, C9, C7, and C1 by cytochrome P450 monooxygenase to form oxetane; the formation of epoxypropane D-ring at C4 and C5; and then CoA acylation to synthesize the key intermediate baccatin III (Croteau et al., 2006). The C13 side chain of taxol is the key group for anticancer activities. Baccatin III is connected with phenylisoserine side chain at C13, and then hydroxylated and benzoylated at C2 and C3 of the side chain to achieve paclitaxel (Howat et al., 2014). Jennewein and coworkers simultaneously overexpressed four genes in *S. cerevisiae* (Engels et al., 2008), including the genes encoding taxadiene synthase, geranylgeranyl diphosphate (GGPP) synthase, HMG1 reductase (thmgr), and the transcription factor UPC2-1. The yield of taxa-4(5),11(12)-diene in the engineered microorganism was 8.7 ± 0.85 mg/L, and geranylgeraniol accumulated at concentrations of up to 33.1 ± 5.6 mg/L (Figure 3, blue route). The Scott team introduced four genes (1-deoxy-d-xylulose 5-phosphate (DXP) synthase, IDP isomerase, GGDP synthase, and taxadiene synthase) to engineer *E. coli* to produce taxadiene for the first time; the authors confirmed that DXP reductoisomerase catalyzes the conversion of DXP to C-methyl-4-erythritol-4-phosphate (Huang et al., 2001). Then, the synthetic genes were divided into an upstream module (*dxs*-*idi*-*ispD*-ispF, containing genes responsible for IPP and DMAPP synthesis) and a downstream module (containing genes responsible for GGPP and taxadiene synthesis). Four genes (*dxs*-*idi*-*ispD*-*ispF*) of the upstream metabolic module were overexpressed by an operon, and the expression of the two genes of the downstream module was regulated by changing the plasmid copy number and the strength of the promoter. Thus, a taxa-4(5),11(12)-diene yield of 1.02 ± 0.08 g/L in *E. coli* (Figure 3, pink route), which was a breakthrough in paclitaxel synthesis, was achieved by synthetic biology (Ajikumar et al., 2010). P450-mediated oxidation is the vital reaction for taxadiene to synthesize baccatin III; thus, two P450 enzymes and an acetyltransferase enzyme were introduced into yeast; subsequent coculture of this yeast and *E. coli* was used to obtain taxadien-5α-acetate-10β-ol yield of 1 mg/L (Figure 3, purple route; Zhou et al., 2015). Low expression and weak catalytic activity of CYPs involved in modification of the taxadiene skeleton are the major stumbling block to further construct the production taxadiene-5α-ol in yeast and *E. coli*. There are often many P450 enzymes involved in the biosynthesis of metabolites (Gnanasekaran et al., 2016), and P450 enzymes of most eukaryotes are membrane bound. Therefore, in the process of constructing and engineering strains full consideration of membrane structure and compartmentalization may be an important aspect to increase yield (Bharati et al., 2012). Wang team employed a compartmentalized metabolic engineering strategy and introduced the genes of taxadiene synthase, taxadiene-5α-hydroxylase, and cytochrome P450 reductase in the chloroplasts of *Nicotiana benthamiana* for the production of taxadiene-5α-ol in tobacco leaves with 1.3 μg/g of fresh weight (Li et al., 2019). Expedient identification of the catalytic elements required for the formation of the D ring and carbonylation of the C-9 position during the synthesis of baccatin III is expected to enable the biosynthesis of 10-desacetylbaccatin III and subsequent chemical synthesis of docetaxel. So far, the epoxidase and oxomutase related to the synthesis of baccatin III have not been cloned and identified. Therefore, for paclitaxel, the first thing to be solved is to elucidate the unclear reactions and the functions of those enzymes in the biosynthetic pathway. In further reconstruction of the biosynthetic pathway, due to the large number of oxidation reactions in the postmodification process, it is an essential and important factor to fully consider the compartmentalized strategy (Du and Li, 2021).

### Assembly of the Precursors of Tanshinone and Breviscapine

*Salvia miltiorrhiza* Bunge (Danshen) is a medicinal plant of the Lamiaceae family. Dried roots of this plant have been used in TCM for a long time because the hydrophilic phenolic acids and lipophilic constituents of these roots are pharmaceutically active components (Wu and Wang, 2012; Xu et al., 2018). Most of the lipophilic constituents, tanshinone I (TS I), TS IIA, and cryptotanshinone (CTS), have multiple pharmacological activities, including anticancer (Kim et al., 2015; Munagala et al., 2015) and antibacterial effects (Yu et al., 2014), and can be efficiently used for the treatment of cardiovascular diseases (Wu and Wang, 2012; Xu et al., 2018). GGPP is the tanshinone precursor in the cytosolic MVA pathway and plastid-localized MEP pathway and is cyclized by the enzymes diterpene synthase copalyl diphosphate synthase (SmCPS) and kaurene synthase-like (SmKSL) to form the diterpenoid skeleton of miltiradiene (Zhou et al., 2012), which is an important precursor for the biosynthesis of tanshinone.

**Figure 4:**
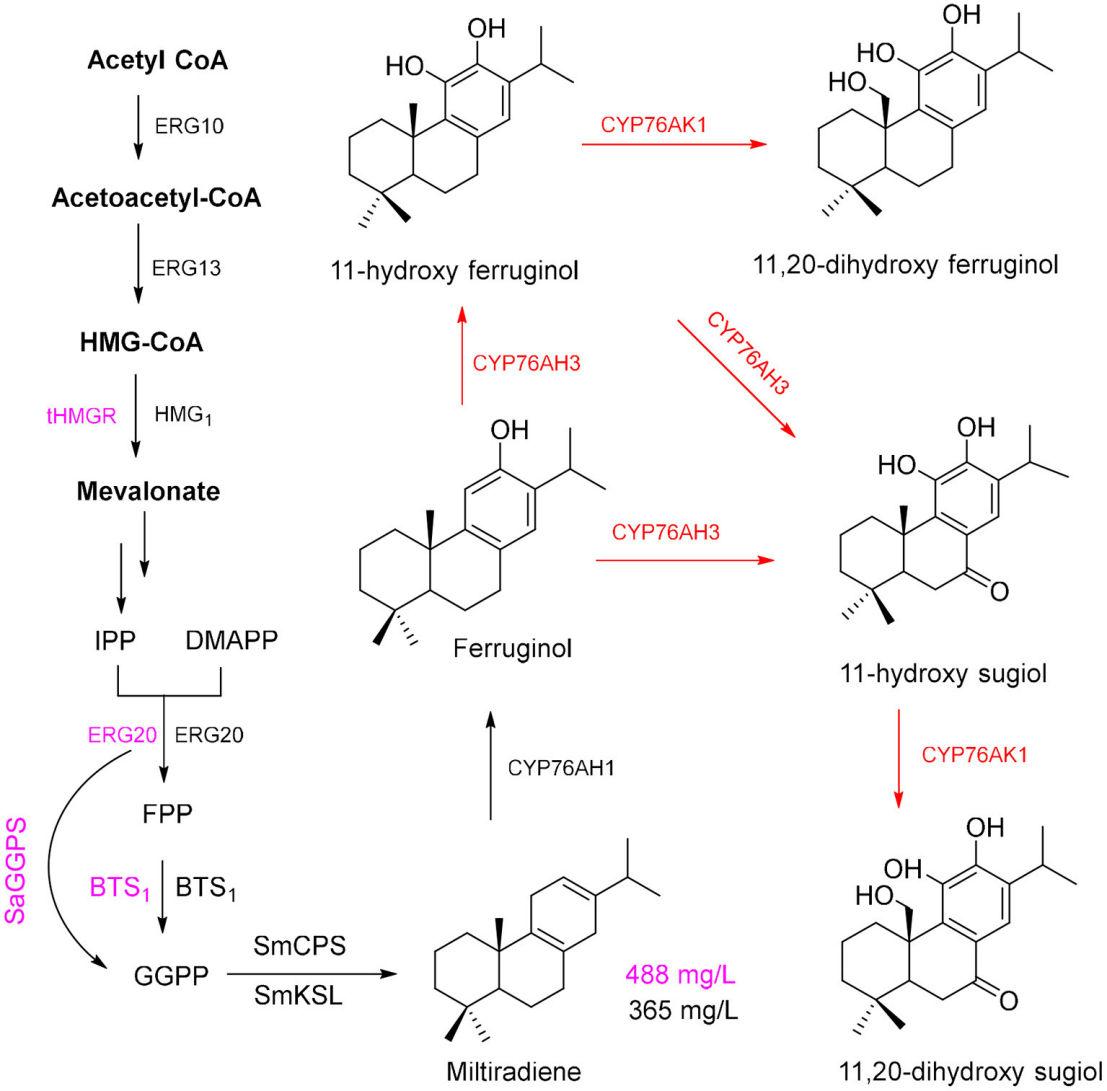
Production route for tanshinone precursors.

Using the modularized pathway engineering techniques, a mutigenes cassette encoding SMCPS, SMKSL, ERG-20, BTS1, and HMG1 was established, and a high yield of miltiradiene (365 mg/L) was obtained in yeast (Zhou et al., 2012). Optimization of the pathway in a yeast expression system (Dai et al., 2012) achieved a miltiradiene yield of 488 mg/L (Figure 4, purple route). By knocking out *UAS* to repress the expression of *ERG9*, knocking out *Rox1* (repressor of hypoxia) transcriptional regulator, and deleting the distant genetic loci *YPL062w* and *YJL064w*, a high-yielding GGPP chassis line was obtained; then, two high-efficiency catalytic enzymes (CfTPS1 and SmKSL1) were constructed to be a SmKSL1-CfTPS1 fusion protein, which can convert GGPP to milradiradiene with the highest efficiency in yeast; finally, the production of milradiradiene was further increased by truncating the N-terminal chloroplast transit peptide (M1-C47) of SmKSL1, and the yield of miltiradiene was increased to 3.5 g/L (Hu et al., 2020). Moreover, some cytochrome P450 monooxygenases (CYP76AH1, CYP76AH3, and CYP76AK1) were identified and functionally characterized to participate in the conversion of miltiradiene to 11,20-dihydroxy ferruginol (Guo et al., 2013, 2016). The subsequent oxidation-reduction steps of the downstream pathway of biosynthesis of tanshinone from 11,20-dihydroxy ferruginol are unclear (Figure 4). Moreover, the conversion of miltiradiene to tanshinone requires multi-oxidation reactions by CYPs (Mizutani and Sato, 2011; Xu et al., 2015). Through transcriptome data, four potential CYP450s may involve in tanshinone biosynthesis compared with the methyl jasmonate induction group (Chang et al., 2019). However, it requires a lot of hard work to identify and elucidate the functions of CYPs in tanshinone biosynthesis.

Breviscapine is a traditional Chinese medicine (TCM) prescription drug obtained from *Erigeron breviscapus* and is used for the treatment of cardio- and cerebrovascular diseases (Gao et al., 2017), which are the main causes of death and disability worldwide (Prince et al., 2015). A gradual increase in the aging population of China has resulted in the growing market demand for breviscapine, and the extraction of *E. breviscapus* is insufficient to meet these needs. Thus, identification of new methods to obtain high amounts of breviscapine is urgently required. Scutellarin and 7-*O*-glucuronide apigenin are the two main precursors of breviscapine; these compounds are generated by glycosylation of apigenin and scutellarein at C-7 (Jiang et al., 2014). The catalysis steps from l-phenylalanine to apigenin were characterized and include six enzymes, namely: phenylalanine ammonia-lyase (PAL), cinnamate-4-hydroxylase, 4-coumaroyl-CoA ligase (4CL), chalcone synthase, chalcone isomerase, and flavone synthase II (Pandey et al., 2016). In 2018, two key genes, namely, *F7GAT* (encoding flavonoid-7-O-glucuronosyltransferase, which converts apigenin into apigenin-7-O-glucuronide) and *F6H* (encoding flavone-6-hydroxylase, which catalyzes C-6 hydroxylation of apigenin and apigenin-7-O-glucuronide), were characterized and engineered to be expressed in combination with the six genes described above in yeast to establish a microbial factory. The yields of scutellarin and apigenin-7-O-glucuronide in yeast factories reached 108 and 185 mg/L, respectively (Liu et al., 2018). Scutellarin is the main active component for clinical usage in *E. breviscapus* (Chledzik et al., 2018). Although in the present work, a considerable yield was achieved, but it was not sufficient for commercial production. It requires to further optimize the metabolic pathway, especially enhancing expression of two genes (encoding PAL and 4CL), which may be the limiting steps in flavonoid biosynthesis (Stahlhut et al., 2015; Nabavi et al., 2020).

### Formation of Two Benzylisoquinoline Alkaloids

Analgesic morphine and antitussive codeine are important medicines for pain management and palliative care. Thebaine is the key benzylisoquinoline alkaloid in the biosynthesis of opium poppy (*Papaver somniferum*) and an important starting material for the semisynthesis of widely demanded drugs (oxycodone, naltrexone, and naloxone) (Ehrenworth and Peralta-Yahya, 2017; Presley and Lindsley, 2018). Recent efforts to reconstitute the production of opiate alkaloids in microbial systems by genetic engineering are expected to alleviate market concerns surrounding plant-based production, including the adverse effects of climate change, pests, and diseases (Tatsis and O'Connor, 2016). Thebaine biosynthesis is complex, and the route starts from 4-hydroxy-phenylacetaldehyde (4-HPAA) and dopamine to generate the primary intermediate (S)-norcoclaurine by norcoclaurine synthase (NCS); then, (*S*)-norcoclaurine is converted to (S)-reticuline via a series of reactions catalyzed by norcoclaurine 6-O-methyltransferase (6OMT), coclaurine *N-*methyltransferase (CNMT), and 4′-O-methyltransferase (4OMT). Then, a critical step of the conversion of (*S*)-reticuline to (*R*)-reticuline is catalyzed by 1,2-dehydroreticuline synthase (DRS) and 1,2-dehydroreticuline reductase (DRR). Salutaridine, the first tetracyclic promorphinian alkaloid, is formed via intramolecular carbon–carbon phenol coupling of (*R*)-reticuline catalyzed by the cytochrome P450 monooxygenase salutaridine synthase (SalSyn). NADPH-dependent salutaridine reductase (SalR) reduces the C7 keto group of salutaridine in a stereospecific manner, yielding salutaridinol, which undergoes stoichiometric transfer of an acetyl group to the C7 hydroxyl moiety by acetyl-CoA–dependent salutaridinol 7-O-acetyltransferase (SalAT) to form salutaridinol-7-O-acetate. Spontaneous loss of the acetyl group results in rearrangement to thebaine, the first pentacyclic morphinian alkaloid. Thebaine is O-demethylated by thebaine 6-O-demethylase (T6ODM) to neopinone, which is spontaneously converted to codeinone (Hirata et al., 2004; Ziegler et al., 2009; Onoyovwe et al., 2013).

**Figure 5:**
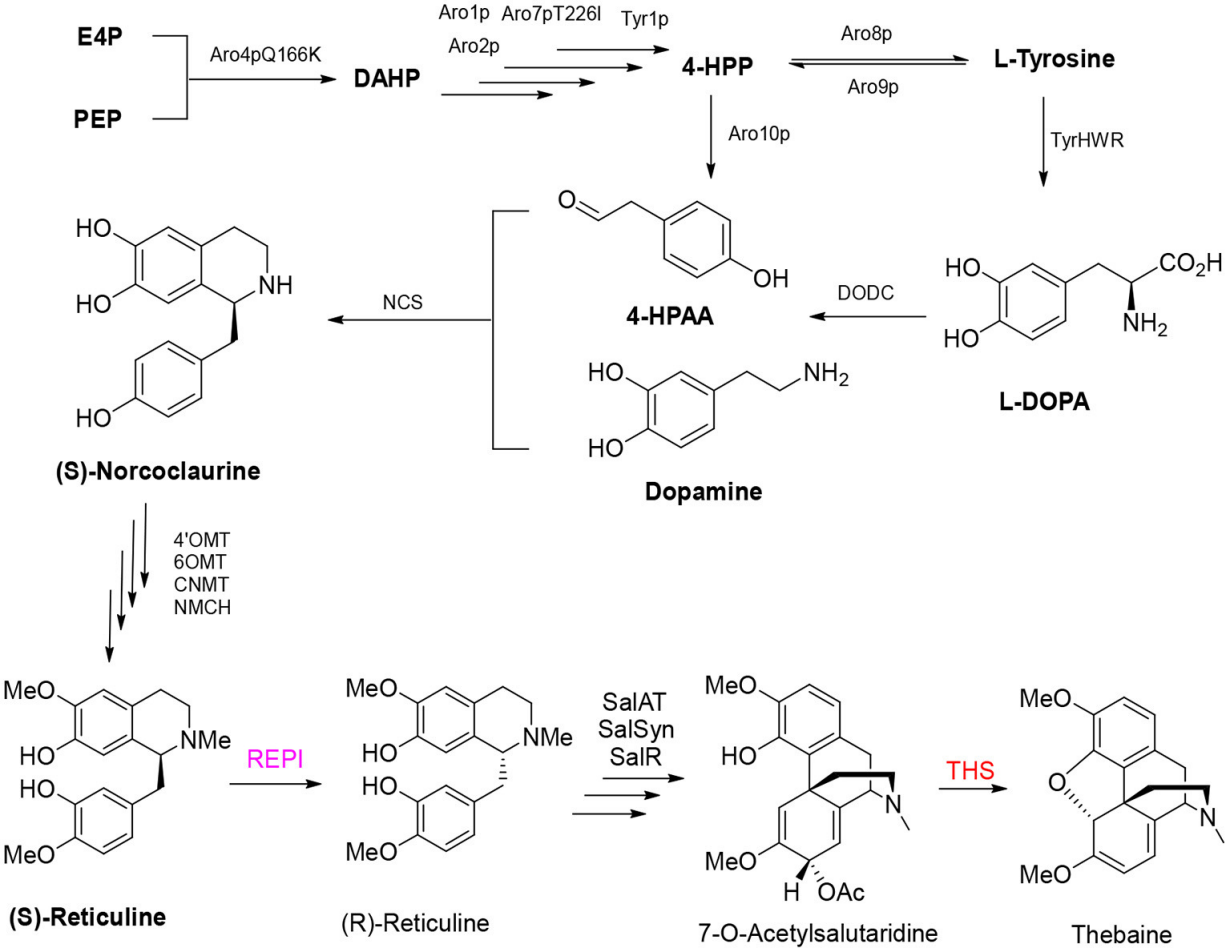
Production of thebaine in engineered yeast.

In 2015, the Galanie team reported their landmark work in yeast to achieve the biosynthesis of opioid compounds (thebaine and hydrocodone) from glucose (Galanie et al., 2015). Successful construction of a thebaine synthesis pathway containing 21 enzymes and a hydrocodone synthesis pathway containing 23 enzymes in yeast involved 24 heterologous expression cassettes, including 21 exogenous enzymes from plants, mammals, and bacteria. Overexpression of two native yeast enzymes and inactivation of a native yeast enzyme resulted in the final titer of thebaine reaching 6.4 ± 0.3 μg/L (Figure 5). The final step in the formation of thebaine has been considered a spontaneous reaction for considerable time (Lenz and Zenk, 1995). However, thebaine synthase (THS) was shown to produce thebaine by the efficient hydroxylation of (7S)–salutaridinol-7-O-acetate, and introduction of the enzyme into engineered yeast increased the yield of thebaine 20-fold (Chen et al., 2018).

Noscapine is another benzylisoquinoline alkaloid (BIA) used as an antitussive drug (Rida et al., 2015); unlike opiate alkaloids, noscapine does not possess analgesic or narcotic properties. All noscapinoids have been isolated from opium poppy, seriously restricting the application of noscapine in the pharmaceutical market due to the negative environmental effects and high cost of large-scale synthesis of noscapine (Chen et al., 2015). The biosynthetic route of noscapine involves 11 enzymatic steps, starting from the key intermediate (*S*)-reticuline, which is a BIA branch-point precursor for the formation of noscapine and morphine (Dang and Facchini, 2012). In 2012, a 10-gene cluster encoding enzymes responsible for the biosynthesis of noscapine from (*S*)-scoulerine was identified in opium poppy (Winzer et al., 2012). The cluster encodes four cytochrome P450 monooxygenases (CYP719A21, CYP82Y1, CYP82X1, and CYP82X2), three methyltransferases (PsMT1, PsMT2, and PsMT3), a carboxylesterase (PsCXE1), a short-chain dehydrogenase/reductase (PsSDR1), and an acetyl transferase (PsAT1), making it one of the most complex natural product pathways described in plants. In 2016, Li et al. engineered a gene cluster in yeast for the production of noscapine, with a yield of 1.64 ± 0.38 μM, starting from the simple alkaloid norlaudanosoline and utilizing 14 biosynthetic steps (Li et al., 2016). This experiment validated the reaction matrix of the final three steps (PsCXE1, PsSDR1, and PsMT2/PsMT3) and suggested that the remaining 4-O-methylation step is catalyzed by two different O-methyltransferases together. Furthermore, this research team combined and introduced more than 30 enzymes from plants, bacteria, mammals, and yeast into chassis cells for efficient synthesis of (S)-reticuline, including 7 endoplasmic reticulum (ER)-localized plant enzymes, by *de novo* production of noscapine in *S. cerevisiae* (Li et al., 2018). Modification and optimization of norcoclaurine synthase and tyrosine hydroxylase increased the content of noscapine 18,000-fold to reach the yield of 2.2 mg/L. Even though this yield of noscapine did not result in commercial production by microorganisms, the microbial biosynthetic platform is expected to promote the discovery and development of alkaloid drugs.

The opium poppy, *P. somniferum*, is the source of codeine and morphine, which accumulate in special cells called laticifers. Morphine, codeine, and thebaine exist in roots and aerial plant parts, and especially accumulate in vesicles in laticifers (Weid et al., 2004; Beaudoin and Facchini, 2014). Several enzymes involved in the biosynthesis of benzylisoquinoline alkaloids are related to subcellular compartments other than the cytosol. For instance, NCS, BBE, and DBOX are presumably located in the lumen of the endoplasmic reticulum (ER); and cytochrome P450 enzymes (NMCH, CFS, SPS MSH, and P6H) are anchored in the cytoplasmic side of the ER, while methyltransferases (6OMT, CNMT, 4'OMT, and TNMT) are contained in the cytosol (Beaudoin and Facchini, 2014; Singh et al., 2019). Subcellular compartmentalization is an evolutionary strategy for dealing with unproductive or harmful crosstalk. In eukaryotes, organelles enclosed by lipid membranes are used to direct enzyme activity to specific substrates. Several benzylisoquinoline alkaloids (e.g., thebaine, noscapine, sanguinarine, and dihydrosanguinarine) were rebuilt the full biosynthetic pathways into microorganisms (Fossati et al., 2014; Trenchard and Smolke, 2015). However, the low yield is far from reaching the expected purpose of using microbial fermentation to produce. Toxicity of heterologous proteins and metabolites is a crucial problem in using synthetic biology to produce natural drugs. The toxicity will lead to slow growth of the host and low content of the final target compound in the fermentation production process. When producing benzylisoquinoline alkaloid by heterologous expression in *S. cerevisiae*, the enzyme NCS that synthesizes (*S*)-norcoclaurine was toxic in the cytosol of host. But high production of benzylisoquinoline alkaloid in heterologous hosts required high NCS activity. The team of Dueber utilized compartmentalization of toxic NCS in the peroxisome, and this alleviates cytotoxicity, thereby increasing the production of benzylisoquinoline alkaloid (Grewal et al., 2021). Therefore, after the complete biosynthetic pathways are characterized at the biochemical and genetic levels, the understanding of enzyme interaction, metabolic regulation, and compartmentalization will be more important challenges for commercial production.

### Biosynthesis of Etoposide

Etoposide is a plant-derived drug and is used in chemotherapy regimens for the treatment of lung cancer, testicular cancer, lymphomas, and other malignancies (Hande, 1998). Etoposide is synthesized starting from two coniferyl alcohols that form pinoresinol in a reaction catalyzed by dirigent protein oxidase (DPO). Pinoresinol is reduced through lariciresinol to form secoisolariciresinol by pinoresinol-lariciresinol reductase (PLR), and this product is subsequently oxidized to matairesinol by secoisolariciresinol dehydrogenase (SDH); then, matairesinol is converted to pluviatolide in a methylenedioxy bridge formation reaction catalyzed by CYP719A23 and CYP719A24 (Marques et al., 2013; Teponno et al., 2016). In 2015, Lau and Sattely (2015) used transcriptome mining in *Podophyllum hexandrum* (mayapple) to identify biosynthetic genes of the podophyllotoxin pathway. The expression of 29-candidate gene combinations in tobacco was used to identify six enzymes (OMT3, CYP71CU1, OMT1, 2-ODD, CYP71BE54, and CYP82D61) required for complete biosynthesis of etoposide aglycone. By coexpression of 10 genes in tobacco, they reconstituted the pathway to (–)-4′-desmethylepipodophyllotoxin (the etoposide aglycone), which is a more direct precursor of etoposide than (-)-podophyllotoxin. At present, the biosynthetic pathway for the production of etoposide aglycone (EA) can be divided into three parts as follows: the pathway of endogenous secondary tobacco metabolite coniferyl alcohol (CA), the pathway from CA to (–)-deoxypodophyllotoxin (DPT), and the pathway from DPT to EA. The products at these levels can be readily distinguished by mass spectrometry; however, a considerable increase in production will be required for facile isolation of pure DPT and other late-stage intermediates. Lau hypothesized that catalyzing CA to (+)-pinoresinol is the limiting step in the biosynthetic process of DPT as the DPT yield increases eight times after adding (+)-pinoresinol to the plant chassis as a precursor (Lau and Sattely, 2015). Eight genes (PAL, C4H, 4CL, HCL, C3H, CCoA-OMT, CCR, and CAD) that enable CA production from intracellular phenylalanine and eight other genes (DIR, PLR, SDH, CYP719A13, OMT3, CYP71CU1, OMT1, and 2ODD) that enable the generation of DPT from CA were coexpressed in tobacco leaves to improve the DPT yield in the tobacco system. The accumulation of DPT reached 4.3 mg/g of dry plant weight (Schultz et al., 2019). Ten percent of essential medicines, as defined by WHO, are derived from natural plant products, a majority of which rely on native plants for production (Davey, 2020). Therefore, it is alternate to use tobacco as a production platform for natural products of medicinal plants. From some successful examples of mass production of target products, rebuilding the biosynthetic pathway of target molecules in microbial chassis cells is an important foundation; subsequently, the optimization of metabolic pathway and search for high-efficiency catalytic enzymes are essential.

## Application of Synthetic Biology for Microbial Drugs

Microbial drugs have become the main rich source of natural drugs due to their novel structures, diverse activities, and short production cycles. More than 70% of antiinfection drugs and 50% of drugs for tumor chemotherapy used in the clinic are directly or indirectly derived from microorganisms (Newman and Cragg, 2012). Microorganisms can produce more target metabolites than plants; however, optimization and rearrangement of biosynthetic gene clusters to form a novel route is required for some target metabolites whose yield cannot be increased in the original strain.

### Heterologous Expression of Epothilone

Epothilone is a macrolide produced by the mycobacterium *Sorangium cellulosum* and has antibacterial and cytotoxic activities (Castro-Alvarez et al., 2018). Epothilone and paclitaxel have similar antitumor mechanisms, and epothilone showed several advantageous characteristics, such as simple structure, increased water solubility, and better anticancer activity compared with those of paclitaxel. Thus, epothilone is widely regarded as a potential antitumor drug (Thomas et al., 2007; Sparano et al., 2010). Epothilone is produced as a secondary metabolite in bacteria; however, its yield is very low due to difficulties in the genetic manipulation of *S. cellulosum*, which greatly limits the application of epothilone in cancer treatment.

In 2000, a 56-kb gene cluster responsible for epothilone biosynthesis was identified; the cluster consists of a loading module, a non-ribosomal peptide synthase module, eight polyketide synthase modules, and a P450 cyclooxygenase enzyme (Gerth et al., 2000; Tang et al., 2000). The Julien team introduced the genes encoding these enzymes into *Streptomyces coelicolor* to achieve heterologous production of epothilones A and B for the first time (Tang et al., 2000). The gene cluster encoding the NRPS/PKS enzyme complex, which catalyzes the biosynthesis of epothilones C and D, consists of six open reading frames (ORFs), namely: epoA, epoB, epoC, epoD, epoE, and epoF. Mutka and coworkers redesigned and rearranged the epothilone gene cluster according to the codon preferences of the *E. coli* host, and the engineered *E. coli* produced epothilones C and D; however, the yield was very low (Mutka et al., 2006). This approach may provide an alternate for the search of novel epothilone analogs. Bian et al. (2017) employed a newly established electroporation technique to insert the complete biosynthetic gene cluster of epothilone into *Burkholderia* K481-B101. The results showed that the yield of epothilone increased by ~75-fold and reached 307 μg/L after optimization of the fermentation conditions and genetic modification of the host bacteria. The yield of epothilone reached 21.8 mg/L after the introduction of the whole biosynthetic gene cluster of epothilone in *Myxococcus xanthus* and optimization of the promoters (Yue et al., 2018). Heterologous expression of epothilone has achieved promising results, but so far, the purpose of large-scale production of epothilone through heterologous expression has not been accomplished. Here are some possible reasons as follows. The research successfully expressed the active proteins of the epothilone gene cluster in *E. coli* without further optimizing the metabolic pathway. In addition, it is unknown whether the expressed proteins and products are toxic to the host. Therefore, the yield was very low in *E. coli*. In *Burkholderia*, despite optimization of the cultural conditions, introduction of the exogenous methylmalonyl-CoA biosynthetic pathway, and overexpression of rare tRNA genes, the epothilone yield has been improved. However, it is possible that *Burkholderia* does not produce abundant secondary metabolites, and thus lacks some endogenous and efficient elements for synthesizing PKS-NRPSs, resulting in a low expression yield in *Burkholderia*. Although the expression of epithilones in *M. xanthus* has obtained promising result, the transcription levels of *epoP, epoC*, and *epoD* were low in this experiment (Yue et al., 2018). Therefore, how to use metabolic regulation to stably and efficiently express the epothilone genes in *M. xanthus* may be a key issue for large-scale production.

### Engineering and Heterologous Expression for Finding New Erythromycin

Erythromycin was initially isolated from the fermentation products of *Streptomyces erythraeus* (synonymous to *Saccharopolyspora erythraea;* McGuire et al., 1952) and is a broad-spectrum macrolide antibiotic used to treat gram-positive bacterial infections. According to literature reports, the industrial production of erythromycin is mainly derived from the strains of *S. erythraea* spp. and its different genetic transformation lines, and the yield of erythromycin reaches 2.0–8.0 g/L (Minas et al., 1998; Karnicar et al., 2016). Increasing bacterial resistance promoted the development of second-generation erythromycin analogs (such as clarithromycin) and third-generation erythromycin analogs (such as telithromycin) by modification and transformation of erythromycin. Erythromycin has a high economic value and considerable potential for new drug development.

The synthetic route of erythromycin contains two steps: polyketide skeleton formation and post-modification in *Streptomyces* (Rawlings, 2001). 6-Deoxyerythronolide B (6-dEB), a macrolide aglycone of erythromycin, is produced in six condensation steps, starting with propionyl-CoA and involving six reactions with methylmalonyl-CoA extender units catalyzed by deoxyerythronolide B synthase (DEBS), which includes the DEBS1, 2, and 3 enzymes (Donadio et al., 1991). Furthermore, 6-deoxyerythronolide B is consecutively converted to erythromycin D by EryF, EryB5, and EryC3. Erythromycin D is the first product with antibacterial activity, representing a branch in the biosynthetic pathway. Erythromycin D is converted by hydroxylase EryK to erythromycin C, which is then methylated by the methylase EryG to generate erythromycin A. Additionally, erythromycin D is converted to erythromycin B by methylation catalyzed by EryG, and erythromycin A is generated via a hydroxylation reaction catalyzed by EryK (Staunton and Wilkinson, 1997; Chen et al., 2008). These compounds are important initial precursors of erythromycin; thus, increasing the levels of propionyl CoA and methylmalonyl CoA are necessary to improve the yield of erythromycins. The Pfeifer group introduced the complete biosynthesis gene cluster of erythromycin in engineered *E. coli* to provide propionyl CoA and methylmalonyl CoA, thereby effectively increasing the yield of erythromycin. Moreover, a MatB-dependent pathway provided (2S)-methylmalonyl-CoA upon the addition of exogenous methylmalonate, which streamlined the routes responsible for the production of two starting erythromycin substrates to eventually produce a novel benzyl-erythromycin analog (Pfeifer et al., 2001; Jiang and Pfeifer, 2013). At present, it has a big advantage to achieve a new type of erythromycin by modifying the erythromycin biosynthetic gene cluster and its heterologous expression, which will also be an important field of drug research in synthetic biology.

## Production Systems for Synthetic Biology

The development of synthetic biology has enabled the use of various systems to express heterologous genes. This technology has become an important approach for the production of target drugs. New clinical studies have shown that natural product drugs have a wide range of applications in the treatment of various diseases. An efficient expression system is required to meet the demands for various recombinant drugs. Currently, there is a lack of a universal chassis for heterologous expression of various types of metabolites. Commonly utilized microbial expression systems mainly include the *E. coli, B. subtilis*, yeast, and filamentous fungal expression systems. The former two systems are the most widely used prokaryotic host bacteria, and the latter two systems are generally used for eukaryotic expression. Understanding the biosynthetic mechanism allows rational selection and utilization of these expression systems for the expression of biosynthetic gene clusters for secondary metabolites.

### Prokaryotic Expression Systems

*Escherichia coli* is a preferred microorganism for the production of recombinant proteins. *E. coli* is mainly used for cloning, genetic modification, and small-scale production, and the mechanisms of these processes have been thoroughly studied. *E. coli* has been widely used in the studies of heterologous medicinal proteins because of its clearly defined genetic background, fast reproduction, low cost, high expression level, stable ^2^H/^13^C/^15^N isotope labeling technology, and wide range of applications (Nuc and Nuc, 2006). However, as a prokaryote, *E. coli* has no regulatory mechanism for eukaryotic gene expression and it lacks the ability to catalyze posttranslational modifications (PTMs), especially modifications catalyzed by cytochrome P450 enzymes, which severely limits its application as a recombinant drug factory. PTMs play important roles in the folding, processing, stability, glycosylation, final biological activity, and immunogenicity of the proteins. A previous study has shown that transfer of the N-linked glycosylation system of *Campylobacter jejuni* to *E. coli* may produce glycosylated proteins (Wacker et al., 2002). Deficiency of the *E. coli* expression system for pharmaceutical protein production was overcome by a series of optimization strategies, such as codon optimization (Burgess-Brown et al., 2008), vector construction (Hayashi and Kojima, 2008), development of a new secretion system to reduce inclusion body formation (Cheng et al., 2017), and self-induction (Briand et al., 2016), to optimize the expression of heterologous proteins in *E. coli* and to determine the best strategy.

*Bacillus subtilis* is an alternate host for the expression and secretion of heterologous proteins in some cases. Comparison with *E. coli* indicates that *B. subtilis* has strong protein secretion ability and does not easily form inclusion bodies. Moreover, *B. subtilis* is not pathogenic and has been used as a cell factory for producing enzymes, vitamins, and functional sugars in the expression systems for over a decade (van Dijl and Hecker, 2013). *B. subtilis* was engineered as a new expression system through the deletion of 36% of its genome, which did not affect cell growth or replication (Reuss et al., 2017). However, *B. subtilis* has certain disadvantages, including unstable vectors and protease secretion. To improve the application of this microbe, target genes were integrated into the host chromosome for expression, and a modular integration plasmid kit was developed (Phuong et al., 2012; Radeck et al., 2013). This approach was clearly successful in *B. subtilis* because the development of a promising *B. subtilis* expression system improved three aspects, such as large-scale genome reduction, elimination of carbon catabolite repression for multiple carbon source coutilization, and cell module engineering for specific product synthesis (Liu et al., 2019).

### Yeast Expression Systems

Yeast is the simplest eukaryotic organism and is thus more suitable than prokaryotes for the expression of active eukaryotic proteins and can accomplish posttranslational modifications, including proteolysis of signal peptides, formation of disulfide bonds, and glycosylation. In particular, yeast is more suitable for gene expression of eukaryotic cytochrome oxidoreductases. *S. cerevisiae* has clear advantages in physiological characteristics related to industrial ethanol production, such as tolerance to pH, high concentrations of ethanol, high sugar content, and high osmotic pressure (Baghban et al., 2019). Currently, many products in the market are produced by using *S. cerevisiae*, including artemisinic acid (Paddon et al., 2013), taxadiene (Engels et al., 2008), noscapine (Li and Smolke, 2016), ginsenoside compound K (Yan et al., 2014), and various types of vaccines (Nielsen, 2013). However, due to low protein yield, high glycosylation of glycoproteins, plasmid instability, and limited amounts of building blocks, the application of commercial products of *S. cerevisiae* is limited. *Yarrowia lipolytica*, a nonconventional yeast, has been extensively investigated and was considered a potential host for the production of natural products (Ma et al., 2020; Muhammad et al., 2020). This yeast has several advantages as an industrial host, including the ability to grow normally on low-cost substrates (e.g., sugars, lignocellulose, fatty acids, fats, waste oils, and crude glycerol) and under high-stress conditions (organic acids, hypersalinity, and metal stresses). Moreover, *Y. lipolytica* efficiently produces key structural building precursors (acetyl–CoA and malonyl–CoA), which are the main carbon sources for terpenoids and polyketides. Moreover, *Y. lipolytica* genes contain more introns than the genes of *S. cerevisiae* (~15 and 4%, respectively), and this yeast has all the known types of alternative splicing (Mekouar et al., 2010), suggesting possible roles for regulated gene expression and/or generation of additional proteome complexity in *Y. lipolytica*. Most intron genes in yeast are not necessary for growth; however, a recent study demonstrated that introns in the yeast genome are independent of the host genes and can promote cell survival under starvation conditions, which may provide new ideas for the construction of new yeast expression systems to increase the production of heterologous proteins (Parenteau et al., 2019).

### Filamentous Fungal Expression Systems

Heterologous expression of a large fragment of the secondary metabolite biosynthetic gene cluster has been performed mainly in the yeast *S. cerevisiae* and in the filamentous fungi *Aspergillus oryzae* and *Aspergillus nidulans*. Filamentous fungi have a very strong ability to express and secrete recombinant proteins and have posttranslational processing functions, such as glycosylation, cleavage by proteases, and the formation of disulfide bonds in heterologous proteins (Meyer, 2008). Many fungal hosts utilized for heterologous expression, such as *A. niger* and *A. oryzae*, are safe and have been employed in the food and food processing industries for a long time; these species have some clear advantages. First, those fungal hosts can express a complete gene cluster of natural fungal product biosynthesis, and the introns can be correctly spliced in the gene cluster; thus, intron removal is not required (Lazarus et al., 2014; Ma et al., 2016; He et al., 2018). Moreover, filamentous fungi can produce target products at low cost. For example, a high yield of multiactive enniatin can be obtained through heterologous expression in filamentous fungi. The nonribosomal polypeptide synthetase gene esyn1 encoding a nonribosomal peptide synthetase for enniatin from *Fusarium oxysporum* was expressed in *A. niger* under the control of the tunable bacterial–fungal hybrid promoter Tet-On. Optimized bioreactor cultivation of *A. niger*, engineered to include strong inducible Tet-On system, produced ~5 g/L enniatin (Meyer et al., 2015). At present, the primary disadvantage of the use of filamentous fungi as expression systems is that most fungi grow into cotton-like hyphal clusters under immersion conditions; these clusters are large spheres that can adhere to the surface of a fermenter and hinder the transfer of the substances and oxygen (Madhavan et al., 2017). On the other hand, filamentous fungi have considerable potential to secrete proteins due to higher number of hyphal branches and hyphal tips (Biesebeke et al., 2005). Long-term applications of filamentous fungi have been developed into a variety of efficient expression systems by means of genetic modification, mutagenesis, and culture optimization. Reasonable use of these promising expression systems may provide an excellent platform for research on biosynthetic pathways of natural products and heterologous expression.

In general, *E. coli*, yeast, and filamentous fungi are used primarily as expression systems. A suitable alternative microbial expression system with similar source genes may be preferentially selected for the production of some post-translationally modified proteins and membrane-bound proteins. For example, the P450 pathway of *Streptomyces* may be selected for expression in *Streptomyces*; the fungal P450 pathway may be selected for expression in filamentous fungi; and the plant P450 pathway may be generally selected for expression in yeast. The P450 enzymes of most eukaryotes are membrane bound, and most of them are located in the endoplasmic reticulum. The N-terminal region of these P450 enzymes contain a transmembrane helix responsible for the anchoring. However, modification at the N-terminus of a plant P450 enzyme enables normal expression in *E. coli* to obtain soluble active protein (Zi and Peters, 2013). The N-terminal anchor is less obstructive in the expression of P450 enzymes in yeast because the yeast intracellular environment is similar to that of plant cells and is more conducive to the expression of plant-derived P450 enzymes. Therefore, *S. cerevisiae* is a highly potent microbial cell factory and a commonly utilized host for the heterologous expression of P450 enzymes.

In addition, the increasing understanding of compartmentalized biosynthesis has promoted the development of compartmentalized engineering to synergize metabolic engineering and enzyme engineering (Heinig et al., 2013; Du and Li, 2021). Compartmental engineering of natural product biosynthetic pathways by changing the subcellular localization of related enzymes has been proven to be an effective strategy for elucidating the biosynthetic mechanism and increasing the titer of the target product. Through the regulation of the metabolic pathways of the compartments, each compartment has a unique physical and chemical environment, which provides favorable conditions for different metabolic pathways; while in subcellular compartments, the local concentration of substrates and enzymes in metabolic pathways may increase, resulting in faster catalytic efficiency (Farhi et al., 2011b); finally, it may be possible to solve the toxicity problem of certain heterologous proteins and metabolites through subcellular compartmentalization (Grewal et al., 2021). Thus, yeast has become a model system for compartmentalization engineering due to the abundant knowledge and genetic tools for manipulating the subcellular localization of enzymes.

## Conclusions

From the limited selection of examples (Table 1), it is obvious that research on the synthetic biology of natural drugs is developing at a rapid and accelerating pace. Research and technological progress in this new field can provide the necessary foundation for the economic viability of the biotechnology industry. It can also be seen from the previous discussion that we have not fully achieved this and there are still many challenges to be solved. For plant natural products, incomplete understanding of biosynthetic pathways is one of the main obstacles preventing pathway reconstruction. For natural products that have reconstructed biosynthetic approach, the yields cannot meet the conditions for large-scale production due to the lack of the knowledge of multienzyme interaction, compartmentalization, or metabolic regulation. In addition, we need to optimize more general hosts to rapidly scale-up the production level of target drugs. If it continues to develop at the current rate, the synthetic biology of natural drugs will quickly move toward mass production and the market.

**Table 1:** Important progresses of selection of examples.

**Targets**	**Host**	**Products**	**Yield**	**References**
Artemisinin	*S. cerevisiae*	Artemisinic acid	100 mg/L	Ro et al., 2006
	*S. cerevisiae*	Amorpha-4,11-diene	40 g/L	Westfall et al., 2012
	*S. cerevisiae*	Artemisinic acid	25 g/L	Paddon et al., 2013
	*N. tabacum*	Artemisinin	6.8 μg/g dry weight	Farhi et al., 2011a
	*N. tabacum*	Artemisinin	0.8 mg/g dry weight	Malhotra et al., 2016
Paclitaxel	*S. cerevisiae*	Taxa-4(5),11(12)-diene	8.7 ± 0.85 mg/L	Engels et al., 2008
	*E. coli*	Taxa-4(5),11(12)-diene	1.02 ± 0.08 g/L	Ajikumar et al., 2010
	co-culture of yeast and *E. coli*	Taxadien-5α-acetate-10β-ol	1 mg/L	Zhou et al., 2015
	*N. tabacum*	Taxadiene-5α-ol	1.3 μg/g fresh weight	Li et al., 2019
Tanshinone	*S. cerevisiae*	Miltiradiene	365 mg/L	Zhou et al., 2012
	*S. cerevisiae*	Miltiradiene	3.5 g/L	Hu et al., 2020
Breviscapine	*S. cerevisiae*	Scutellarin	108 mg/L	Liu et al., 2018
Thebaine	*S. cerevisiae*	Thebaine	6.4 ± 0.3 μg/L	Galanie et al., 2015
Noscapine	*S. cerevisiae*	Noscapine	2.2 mg/L	Li et al., 2018
Etoposide	*N. tabacum*	Deoxypodophyllotoxin	4.3 mg/g dry weight	Schultz et al., 2019
Epothilone	*Burkholderia* K481-B101	Epothilone	307 μg/L	Bian et al., 2017
	*M. xanthus*	Epothilone	21.8 mg/L	Yue et al., 2018
Erythromycin	*E. coli*	Novel benzyl-erythromycin	–	Jiang and Pfeifer, 2013

## Author Contributions

C-QL and P-JZ wrote the main part of the manuscript. H-ML, Q-YH, and G-HL made significant contributions and particularly in revising the manuscript. C-QL, G-HL, and P-JZ prepared and formatted the references. All authors contributed to the manuscript and approved the submitted version.

## Conflict of Interest

The authors declare that the research was conducted in the absence of any commercial or financial relationships that could be construed as a potential conflict of interest.

## Publisher's Note

All claims expressed in this article are solely those of the authors and do not necessarily represent those of their affiliated organizations, or those of the publisher, the editors and the reviewers. Any product that may be evaluated in this article, or claim that may be made by its manufacturer, is not guaranteed or endorsed by the publisher.
